# Women’s Occupational Tobacco Dust Exposure in Indonesia (T-CHARM): Protocol for a Prospective Cohort Study

**DOI:** 10.2196/84231

**Published:** 2025-12-31

**Authors:** Ancah Caesarina Novi Marchianti, Athira Nandakumar, Elvia Rahmi Marga Putri, Dwita Aryadina Rachmawati, Sugiyanta Sugiyanta, Hairrudin Hairrudin, Elly Nurus Sakinah, Jauhar Firdaus, Muflihatul Muniroh, Eka Djatnika Nugraha

**Affiliations:** 1 Department of Public Health Faculty of Medicine University of Jember Jember Indonesia; 2 Department of Epidemiology and Preventive Medicine Graduate School of Medical and Dental Sciences Kagoshima University Kagoshima Japan; 3 Department of Clinical Pathology Faculty of Medicine University of Jember Jember Indonesia; 4 Department of Biochemistry Faculty of Medicine University of Jember Jember Indonesia; 5 Department of Pharmacology Faculty of Medicine University of Jember Jember Indonesia; 6 Department of Physiology Faculty of Medicine University of Jember Jember Indonesia; 7 Department of Physiology Faculty of Medicine Diponegoro University Semarang Indonesia; 8 Center for Clinical Toxicology and Environmental Health Faculty of Medicine Diponegoro University Semarang Indonesia; 9 Research Center for Safety, Metrology, and Nuclear Quality Technology Research Organization for Nuclear Energy National Research and Innovation Agency South Tangerang Indonesia

**Keywords:** tobacco dust, women health, preventive medicine, occupational disease, industrial hazard

## Abstract

**Background:**

The consumption of tobacco is regarded as a contributing factor to several diseases. However, the impact of tobacco dust exposure (TDE) on tobacco workers has not been extensively investigated.

**Objective:**

This protocol introduces the design and implementation of the Tobacco Dust Cohort for Health Assessment and Risk Monitoring (T-CHARM) study, a prospective cohort study aimed at evaluating the health impacts of TDE.

**Methods:**

This prospective cohort study will recruit women working in tobacco processing who are nonsmokers and women who do not work for the tobacco industry and are nonsmokers living in a nearby area (unexposed group), with a total of 400 expected participants. The impact of TDE on health, including metabolic syndrome parameters; complete blood count; and cardiovascular, liver, renal, and lung function, will be evaluated in relation to urine cotinine levels. Air quality and chemical substances in the air and leaves will also be analyzed. The data will be subsequently analyzed using appropriate statistical tools.

**Results:**

A total of 120 respondents have participated as of August 2025. Another 80 respondents will be recruited, laboratory analysis is ongoing, and baseline results are expected by the end of 2025.

**Conclusions:**

The strength of the T-CHARM study’s approach is its detailed occupational and environmental factors and longitudinal health data from the corporate clinic or the district health center, as well as links to cancer and mortality registries and self-reported health. The current phase of the study focuses on baseline data collection for long-term follow-up. The cohort will be monitored for up to 20 years, depending on sustained funding. T-CHARM offers a robust framework for understanding the chronic health effects of occupational TDE.

**International Registered Report Identifier (IRRID):**

DERR1-10.2196/84231

## Introduction

The tobacco industry is one of the biggest industries and provides millions of jobs [1]. In Indonesia, Jember Regency has long been recognized as “Tobacco City,” reflecting its deep-rooted economic and cultural ties to tobacco cultivation and processing [2]. While most research on tobaccosis (chronic tobacco poisoning) has focused on people who smoke, there is a gap in studies examining occupational exposure, particularly among workers in tobacco processing environments who do not smoke [3]. The health effects of tobacco dust exposure (TDE), especially from nicotine-laden particulates, remain poorly understood despite growing concern [4]. This lack of data is critical, as workers may experience chronic exposure without direct tobacco use. Addressing these issues is complex, as the tobacco industry intersects with economic welfare, government policy, and political influence, making regulatory and public health interventions particularly challenging [5,6].

A study was conducted to investigate the concentration of tobacco dust in a tobacco factory in Thessaloniki, Greece. The findings revealed a markedly elevated level of total suspended dust in the workplace, with concentrations ranging from 45.3 to 54.4 μg/m^–3^ [7]. Nicotine has been demonstrated to elevate both blood pressure and heart rate, and to induce atherogenesis in coronary artery endothelial cells [8]. This is attributed to its sympathomimetic effects, which result in an increased heart rate, myocardial contractility, elevated coronary vascular resistance, and decreased insulin sensitivity. Consequently, there is an increased risk of developing cardiovascular diseases, including coronary heart disease and atherosclerosis [9].

This research builds on a 2015 study that yielded notable insights into the impact of TDE on worker well-being [4]. The issue of workers’ health is frequently overlooked due to a lack of knowledge and the absence of the requisite infrastructure, with the health of workers in the tobacco industry being of particular concern. Further evaluation is required to ascertain the effects of chronic TDE, which will be conducted using a prospective cohort study design. We will use an observational design with a cross-sectional method as a preliminary baseline for a prospective cohort study. A future cohort study is anticipated to ascertain the definitive effects of TDE, circumventing the inherent biases of cross-sectional research [10]. The nature of TDE exposure shares similarities with thirdhand smoke, the residual tobacco pollutants that linger on surfaces and in dust, posing risks even in the absence of active smoking. Therefore, the findings from this study are expected to benefit not only tobacco workers but also individuals exposed to thirdhand smoke in residential and occupational settings.

## Methods

### Study Design

The Tobacco Dust Cohort for Health Assessment and Risk Monitoring (T-CHARM) study examines how occupation and the environment of an agricultural area (especially tobacco) can cause diseases and affect people’s well-being over time.

**Figure 1:**
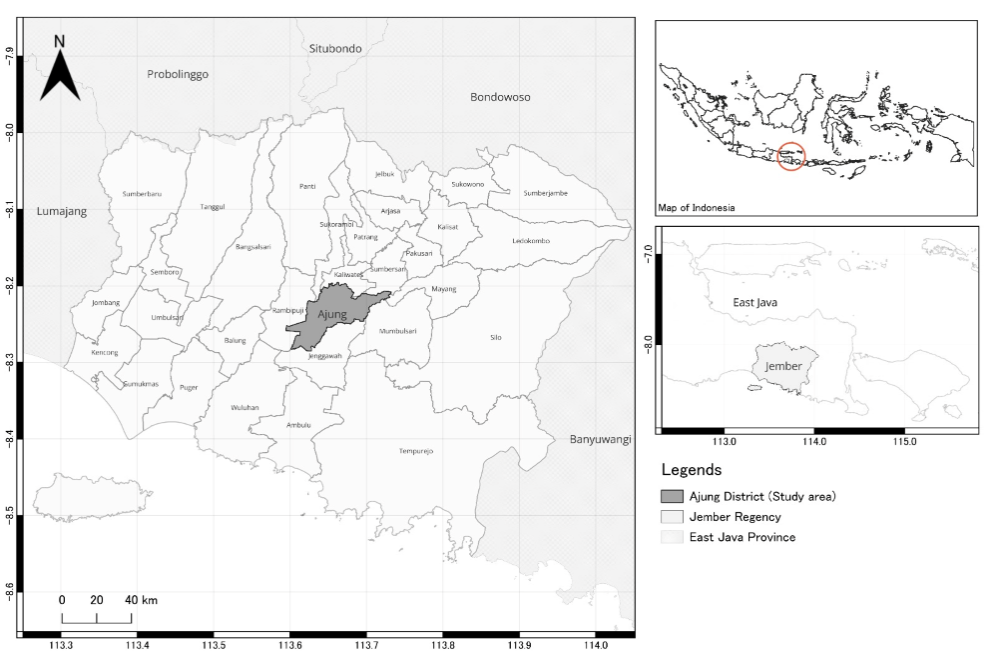
Geographic location of study groups in the Tobacco Dust Cohort for Health Assessment and Risk Monitoring (T-CHARM) cohort. This map illustrates the research location of the T-CHARM study in Ajung District, Jember Regency, East Java, Indonesia. The gray-shaded area represents Ajung District, where both the exposed group (nonsmoking women tobacco processing workers) and the unexposed group (nonsmoking women workers from nontobacco occupations) were recruited.

Starting in 2024, the T-CHARM cohort is based on a tobacco factory and its surrounding areas under the jurisdiction of the Ajung Health Center in Jember, Indonesia (Figure 1). The study is led by local research teams from a regional university in collaboration with international partners from Kagoshima University (Japan), the University of Occupational and Environmental Health (Japan), and the National Research and Innovation Agency (Indonesia).

The project is supported by a range of funding sources, including Directorate of Research and Community Service (Direktorat Penelitian dan Pengabdian kepada Masyarakat), Indonesia; Japan’s Ministry of Education, Culture, Sports, Science and Technology; and institutional grants and travel programs from participating universities in both Indonesia and Japan.

### Study Population and Eligibility Criteria

The T-CHARM study recruited participants from one of the largest tobacco processing industries in Jember, Indonesia, which employs approximately 2333 women in its processing section and is certified by international occupational and safety standards.

#### Exposed Group

Participants in the exposed group are women who work in tobacco processing who are nonsmokers. These individuals are regularly exposed to tobacco dust and related particulates through their occupational activities.

#### Unexposed Group

The comparison group consisted of women who are nonsmokers and employed in occupations unrelated to tobacco within the same district as the tobacco factory. This geographic proximity helps to control for environmental factors unrelated to occupational exposure.

#### Inclusion Criteria

Participants were eligible if they met the following criteria:

- Woman aged 18-55 years
- Nonsmoker (confirmed by self-report and urine cotinine screening)
- Actively employed as laborers for ≥8 hours per day, 6 days per week
- Healthy at the time of recruitment
- Provided written informed consent after screening for eligibility

#### Exclusion Criteria

Participants were excluded for the following reasons:

- Presented with fever or diarrhea, as determined by body temperature screening
- Had any condition that could confound biomarker or health assessments

### Data Collection Method

#### Overview

Baseline data for the T-CHARM study were collected through direct, face-to-face interviews conducted by researchers, with support from physicians and trained surveyors. Data collection took place at the tobacco factory and associated workplaces. A structured questionnaire was administered, comprising closed-ended questions across multiple domains, including sociodemographic information, duration of employment, medical history, smoking status, menstrual history [11], hydration status, ergonomic concerns [12], physical activity levels [13], sleep quality [14], anxiety potential [15], and tobacco exposure assessment.

**Figure 2:**
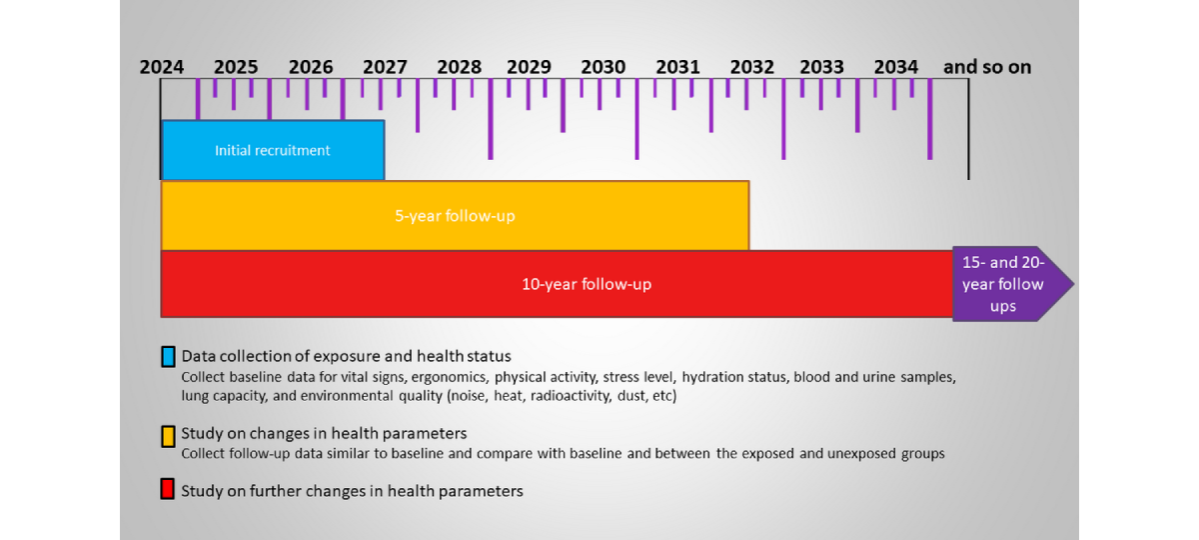
Assessment and data collection schedule.

In addition to the questionnaire, a series of clinical and physiological examinations were performed to evaluate the general health status of participants. These included vital signs assessment [16], BMI calculation, ergonomic observation [17], pulmonary function testing using a portable spirometer (Contec SP10, Contec Medical System Co, Qinhuangdao, Hebei, China) [18], and venous blood sampling (3 mL) for serum analysis, including complete blood count, blood glucose, lipid profile, liver function, and renal function tests. Furthermore, a random urine sample was collected in a sterile container to analyze urine profile and cotinine levels, adjusted for creatinine concentration, using an enzyme-linked immunosorbent assay. To monitor longitudinal changes in health parameters, the same data collection protocol will be repeated every 5 years over 20 years (Figure 2).

#### Blood Examination Procedures

An automatic hematology analyzer is used to examine the complete blood count [19,20]. A small aliquot of a well-mixed blood is placed into the hematology analyzer sample holder. The analyzer uses optical and electrical methods (eg, Coulter principle) to count and characterize blood cells, including red blood cells, white blood cells, hemoglobin, hematocrit, and platelets. The reaction happens inside chambers where cells are lysed or stained as needed for measurement. The analyzer then calculates and displays complete blood count parameters such as red blood cell count, white blood cell count, hemoglobin concentration, hematocrit, platelet count, and red blood cell indices (mean corpuscular volume, mean corpuscular hemoglobin, mean corpuscular hemoglobin concentration).

An enzymatic UV test is conducted using hexokinase to examine fasting blood glucose [21]. Glucose is phosphorylated by hexokinase in the presence of adenosine triphosphate to form glucose-6-phosphate, which is then converted in the presence of NAD^+^ by glucose-6-phosphate dehydrogenase to gluconate-6-phosphate and NADH+H^+^. The increase in absorbance of NADH+H^+^ is determined spectrophotometrically at a wavelength of 340 nm as the end point measurement. Lipid parameters are assessed using standardized enzymatic methods:

- High-density lipoprotein cholesterol and total cholesterol: measured via direct enzymatic homogeneous assays in accordance with International Federation of Clinical Chemistry and Laboratory Medicine guidelines
- Low-density lipoprotein cholesterol: calculated using the Friedewald formula [22]
- Triglycerides: determined by a colorimetric enzymatic test using glycerol-3-phosphate oxidase

#### Urine Examination Procedures

Urine specimens are stored at –20 °C until analysis. Before testing, frozen samples are thawed to room temperature and thoroughly mixed to ensure homogeneity. The cotinine kit is procured from Calbiotech, El Cajon, California, and is a solid-phase competitive enzyme-linked immunosorbent assay. In this method, the sample and cotinine enzyme conjugate are added to the wells coated with anticotinine antibody. Cotinine in the samples competes with a cotinine enzyme (horseradish peroxidase) conjugate for binding sites [23]. The unbound cotinine and cotinine enzyme conjugate are removed by washing. Upon the addition of the substrate, the intensity of the color is inversely proportional to the concentration of cotinine in the sample. The cutoff level of urinary cotinine is considered to be 10 ng/mL.

#### Environment Examination Procedures

##### Air Quality Monitoring

Air quality parameters—including humidity, temperature, and other site-specific conditions—are measured inside the tobacco company to ensure compliance with standards set by the World Health Organization and the Indonesian Ministry of Health [24].

##### Inhalation Exposure Assessment

Inhalation exposure measurements involve radon, thoron, and thoron progeny. Radon (^222^Rn) and thoron (^220^Rn) concentrations are measured with a RADUET monitor (Radosys Ltd, Budapest, Hungary). The RADUET has solid-state track detectors and a thoron monitor. Radon, thoron, and thoron progeny measurements are carried out with indoor and outdoor ambient dose rate measurements. The CR-39 is chemically etched for 24 hours in 6 M NaOH solution at 60 °C to count the tracks, and the radon and thoron concentrations are calculated. The thoron progeny is measured with a stainless steel plate, CR-39, and an aluminized film. Diurnal exposure variation will also be measured with an active detector. A type ^222^Rn monitor (RAD 7, Durridge Co, Billerica, Massachusetts) measures the samples and detects alpha activity for three 24-hour periods for a short-term measurement [25].

#### Particulate Matter Sampling and Analysis

Particulate matter (PM) is collected and analyzed to determine concentration and chemical composition:

- Sampling method: The 4-stage impactor is used to collect samples over 48 hours; PM fractions include total suspended particulates, PM_1_, PM_2.5_, and PM_10_.
- Analytical techniques: Inorganic constituents will be analyzed using Inductively Coupled Plasma Mass Spectrometry (ICP-MS/MS; Perkinelmer, Shelton, Connecticut), and cation and anion speciation is performed via ion chromatography (Thermo, Germany) [26].

#### Bioindicator Sampling

Tobacco leaves are also collected to study the accumulation of airborne pollutants, particularly radionuclides and heavy metals, as bioindicators of environmental exposure.

**Figure 3:**
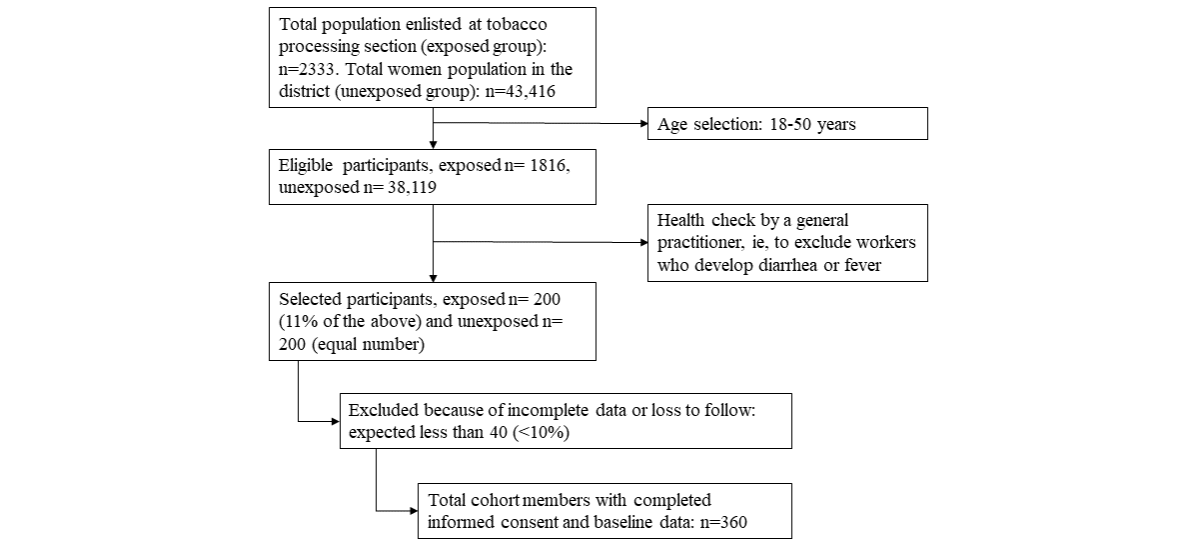
Flowchart of recruitment and participation.

Figure 3 illustrates the recruitment process for the T-CHARM study. The exposed group consisted of a total population of 2333 women who work in tobacco processing, of whom 1816 met the eligibility criteria following screening. From this eligible pool, a target sample of 200 participants was estimated and recruited. To ensure comparability, the unexposed group—women who were nonsmokers and residents of nearby communities—was also designed to include 200 participants, matched by key demographic and environmental factors. The total cohort size for follow-up is projected to reach 400 participants, with data collection encompassing baseline and longitudinal health metrics. All collected data are subjected to analysis using appropriate statistical tools, as outlined in the study’s analysis plan.

### Ethical Considerations

The T-CHARM study received ethical approval from the Institutional Ethical Board in Jember, Indonesia, in July 2024 (2883/UN25.1.10.2/KE/2024) and from the Institutional Ethical Board in Kagoshima, Japan, in September 2024 (240076). Both approvals adhere to the Guideline for Ethical Clearance of Human Research and ensure compliance with national and international standards for research involving human participants. Subsequent continuation of the study was approved by the Faculty of Medicine Ethics Committee under approval number 1578/UN25.1.10.2/KE/2025.

All participants were fully informed of the study’s purpose, procedures, potential risks, and their right to withdraw at any time without consequence; screened according to predefined inclusion and exclusion criteria; and provided written informed consent before enrollment. These procedures ensure that participant autonomy, safety, and confidentiality are upheld throughout the study.

### Data Analysis Plan

All analyses for the T-CHARM study will be conducted using the latest version of Stata (StataCorp). Data entry and cleaning will be performed by trained personnel using a double-entry verification system to ensure data integrity.

#### Missing Data Handling

For missing outcome data at follow-ups, the last observation carried forward method will be applied. Sensitivity analyses will be conducted to evaluate the robustness of findings under alternative missing data assumptions, including complete case analysis and multiple imputations if appropriate.

#### Outlier Management

Outliers in biomarker measurements will be identified using the IQR method. Outliers will be retained unless a confirmed analytic error or sample contamination is identified. Results will be assessed with and without outliers to determine their influence on statistical outcomes.

#### Group Comparisons

Comparative analyses between the exposed and unexposed groups will be performed for respondent characteristics, health examination results, medical history, lung function, and blood and urine parameters. Statistical tests will include Student *t* test for continuous (ratio) variables and Pearson *χ*^2^ test or Fisher exact test for categorical variables, and *P* values <.05 will be considered statistically significant.

#### Exploratory Subgroup Analyses

To minimize bias and explore potential effect modifiers, subgroup analyses may be stratified by BMI, age, and length of employment. These stratified analyses will help identify differential health impacts and refine risk estimates across population subgroups.

## Results

The T-CHARM study was officially funded in the year 2024, with data collection commencing in July 2024. As of August 2025, the study has successfully enrolled 60 participants in each group—women who are nonsmokers and work in tobacco processing (exposed group) and women who are nonsmokers and community members (unexposed group). Recruitment for an additional 80 participants is currently underway, alongside laboratory analyses for the second phase of baseline data collection. These analyses include biomarker profiling, clinical assessments, socioeconomic status, and environmental exposure measurements. The cohort baseline results are expected to be published by the end of 2025, marking a key milestone in the study’s longitudinal design and setting the foundation for future follow-up waves.

In general, among the 60 women recruited for the exposed group, most of them were obese (n=28, 46.7%) and overweight (n=11, 18.3%), 76.7% (n=46) were of Madurese ethnicity, 53.3% (n=32) had education in elementary school, and 45.0% (n=27) were educated until middle and high school. Their mean age was 40 (range 21-52) years. Compared to the other work in that area, tobacco workers are paid well, with 76.7% (n=46) having an income as high as 875,000 IDR (US $52.50). Another key finding is that all the study participants are nonsmokers but exposed to tobacco dust occupationally, and 57.1% (n=60) reported being passive smokers. Health questionnaire answers showed that 60 people complained of eye problems, 28 had dermatological issues, 5 had respiratory diseases, 11 had cardiovascular disease, 60 had neurological problems, and 37 had reproductive issues.

## Discussion

### Study Implications

Most studies related to tobaccosis use active smokers as participants [27,28]. In Indonesia, there has been no research collecting data on tobacco-related diseases, including in the workplace, so the effects of exposure to nicotine-containing tobacco dust on workers’ health are unknown and have not been studied [4]. Problems in this industry are also not easy to solve because they are often related to public welfare, government policy, and sometimes political influence [29]. Previous studies have found significant effects of tobacco dust on tobacco industry workers [4]. Further evaluation is needed to determine the effects of chronic exposure to tobacco dust. This study serves as a baseline for a future cohort study. Further evaluations are expected to determine the effects of tobacco dust, avoiding the biases in cross-sectional studies. This exposure is similar to thirdhand smoking conditions, so the results of this study will benefit those exposed to tobacco at work and through thirdhand smoke in the wider community.

Tobacco leaf sorters play a critical role in determining the appropriate use of tobacco leaves, whether as wrappers or binders, based on their quality. Leaf inspection involves both internal assessments (eg, human sensory evaluation, smoking tests, and chemical analysis) and external examinations (primarily visual inspection) [30]. Workers sort tobacco leaves by evaluating attributes such as quality, size, color, and dryness, requiring sustained attention to fine details over extended periods. This meticulous and repetitive visual task places workers at elevated risk for eye fatigue or asthenopia, particularly in the absence of proper environmental controls and scheduled rest periods [31].

Itching is a common health complaint among new tobacco leaf sorters, often presented as a mild form of contact dermatitis resulting from exposure to nicotine and other chemical residues on the leaves [32,33]. Preventive measures such as the use of personal protective equipment and maintaining proper skin hygiene can significantly reduce the risk of dermatitis [34,35]. However, implementing personal protective equipment—particularly gloves—poses a practical challenge. Sensory assessment of tobacco leaves, which relies heavily on tactile feedback, often requires workers to sort without gloves, limiting the feasibility of full protection.

Tobacco leaf sorters are at risk of developing neurological symptoms such as paresthesia, headaches, and dizziness, which are commonly linked to green tobacco sickness, a form of acute nicotine poisoning caused by transdermal absorption of nicotine from wet tobacco leaves [36,37]. The likelihood of absorption increases significantly when leaves are damp due to dew, rain, or sweat, particularly during early morning or humid working conditions.

Even when individuals are not employed during pregnancy, nausea experienced during pregnancy may be associated with residual effects of prior green tobacco sickness exposure, suggesting potential long-term or latent impacts. To mitigate these risks, it is essential to implement comprehensive preventive strategies aimed at minimizing direct skin contact with tobacco leaves. This includes the use of protective clothing and gloves, although such measures present practical challenges. Gloves, for instance, may interfere with the sensitivity required for leaf sorting, necessitating a careful balance between occupational safety and task performance [38].

### Strengths and Limitations

The goal is to understand how TDE affects health over a person’s lifetime. At enrollment, the T-CHARM study collects demographic and occupational data (eg, current address, job role); lifestyle factors (eg, smoking status, dietary habits); biomarkers of exposure, including urine cotinine and other relevant indicators; and general health assessments, covering cardiovascular, respiratory, hepatic, renal, and metabolic parameters. Follow-up assessments will update exposure metrics and health status periodically, enabling dynamic tracking of disease progression and risk factors [39].

T-CHARM’s major asset is its multisource medical data integration, which includes clinical records from corporate clinics and district health centers; national health insurance data, offering continuity and completeness; and direct input from general practitioners affiliated with the research team, allowing for nuanced clinical insights beyond standard registry linkages. This approach mitigates common limitations in cohort studies, such as recall bias and loss to follow-up from self-reported questionnaires.

While traditional epidemiological studies focus on cancer, respiratory, and cardiovascular diseases, T-CHARM broadens the scope to include neurological disorders, degenerative diseases, and chronic morbidity patterns linked to environmental and occupational exposures. The longitudinal nature of general practitioner data enables detailed tracking of disease onset, progression, and comorbidities—offering a more comprehensive understanding of health trajectories in exposed populations.

Existing biases or weaknesses have been minimized, but as a cohort study, there are some limitations. The limitations include changes in characteristics of participants over time, loss to follow-up, and confounding variables that complicate data analysis due to a lack of randomization. Potential respondents are also sometimes difficult to recruit due to the invasive nature of blood collection. Additionally, biases can arise from knowledge of exposure status, participant dropout, changes in behavior due to participation, and data quality issues in retrospective studies.

### Conclusion

The T-CHARM study is currently in the baseline data collection phase, with plans for long-term follow-up over the next 20 years, contingent on future funding availability. To promote scientific collaboration and maximize the utility of the cohort, external researchers are invited to submit proposals for noncommercial research using the available T-CHARM data. Researchers may also request additional data collection contingent on appropriate funding support. All proposals will be reviewed by the T-CHARM Management Committee to ensure alignment with the study’s objectives and ethical standards. Requests should be directed to the corresponding author, and a data management fee will apply to cover administrative and technical costs associated with data preparation and transfer.

## Appendix

Multimedia Appendix 1 Peer-review reports.

## Abbreviations

220Rn: thoron
222Rn: radon
ICP-MS/MS: Inductively Coupled Plasma Mass Spectrometry
PM: particulate matter
T-CHARM: Tobacco Dust Cohort for Health Assessment and Risk Monitoring
TDE: tobacco dust exposure

## Data Availability

The datasets generated or analyzed during this study will be made available from the corresponding author upon reasonable request.
