# Supplementary material for: Women’s Occupational Tobacco Dust Exposure in Indonesia (T-CHARM): Protocol for a Prospective Cohort Study
Source: JMIR Res Protoc. 2025 Dec 31;14:e84231. doi: 10.2196/84231 (PMC12805322; doi:10.2196/84231)
Supplement: Multimedia Appendix 1 [file resprot_v14i1e84231_app1.pdf]

REVIEW PROPOSAL

- Nama Tim Peneliti : dr. Ancah Caesarina Novi Marchianti, Ph.D.
- Judul : Kedokteran Pencegahan: Kajian Implikasi Jangka Panjang Paparan Debu Tembakau pada Pekerja (Model Third Hand Smoking)

1. Hasil Penilaian

No	Kriteria	Skor * (1, 2, 3 atau 4)
**Kriteria Umum**		
1.	Relevansi dengan bidang keahlian Program Studi (PTM/PTO)	4
2.	Originalitas gagasan penelitian	3
3.	Urgensi/kemendesakan untuk dilaksanakan penelitian dilihat dari masalah yang akan dipecahkan	3
4.	Fisibilitas (keterlaksanaan) penelitian	4
**Kriteria Khusus (Kualitas Proposal)**		
5.	Kesesuaian latar belakang dengan masalah penelitian	4
6.	Ketepatan rumusan masalah	4
7.	Relevansi kerangka kajian pustaka dengan masalah dan tujuan penelitian (termasuk relevansi dan kemutakhiran rujukan yang digunakan)	3
8.	Ketepatan metode dengan masalah dan tujuan penelitian	4
9.	Kesesuaian tata tulis dan format proposal dengan ketentuan yang berlaku	3
**Jumlah Skor**		
**Nilai (Jumlah Skor/36 x 100)**		

LAYAK / ~~TIDAK LAYAK~~** untuk dilanjutkan menjadi penelitian dan didanai***.

2. Saran Revisi

Reviewer 1: Penelitian ini layak untuk didanai. Tujuan dan Rumusan Masalah sudah jelas. Roadmap sudah mencakup penelitian sebelumnya yang berkaitan dengan penelitian saat ini dan yang akan datang.

Reviewer 2: proposal yang diajukan memiliki kebaruan dan pendekatan baru sehingga proposal ini layak didanai. Harap memastikan semua prosedur dan protokol penelitian dilakukan dengan baik dan benar untuk hasil yang akurat.

Reviewer 3: hasil review: isi proposal menarik, gambar roadmap dapat diperjelas dengan topik penelitian yang pernah dilakukan oleh tim pengusul sebagai dasar penelitian saat ini dan penelitian akan datang. pada metode kurang akurat karena tidak menyebutkan tanda vital medis yang diepriksa apa saja karena ini terkait dengan RAB yang diajukan. Pada RAB seharusnya biaya pemeriksaan darah (trigliserid, kolesterol gulad arah dll) bukan dimasukan di bahan karena tidak membeli bahan tetapi biaya pemeriksaan, masuk ke dalam pengumpulan data atau analisis sampel.

(Reviewer)

*) 4 : Sangat Sesuai ; 3 : Sesuai ; 2 : Cukup Sesuai ; 1 :Kurang Sesuai
**) Coret yang tidak perlu
***) Dinyatakan LAYAK apabila Nilai ≥ 70,0 dan tidak ada skor 1

Funded and reviewed in 2025, screenshot proof in BIMA (Research and Community Service Information Base) website of the Indonesian Ministry of Education, Culture, Research, and Technology

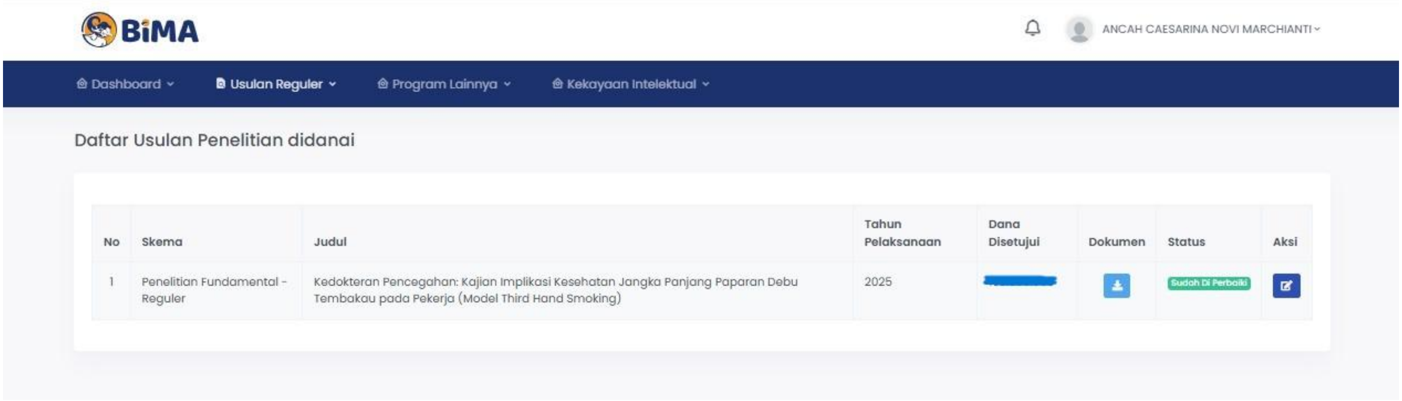

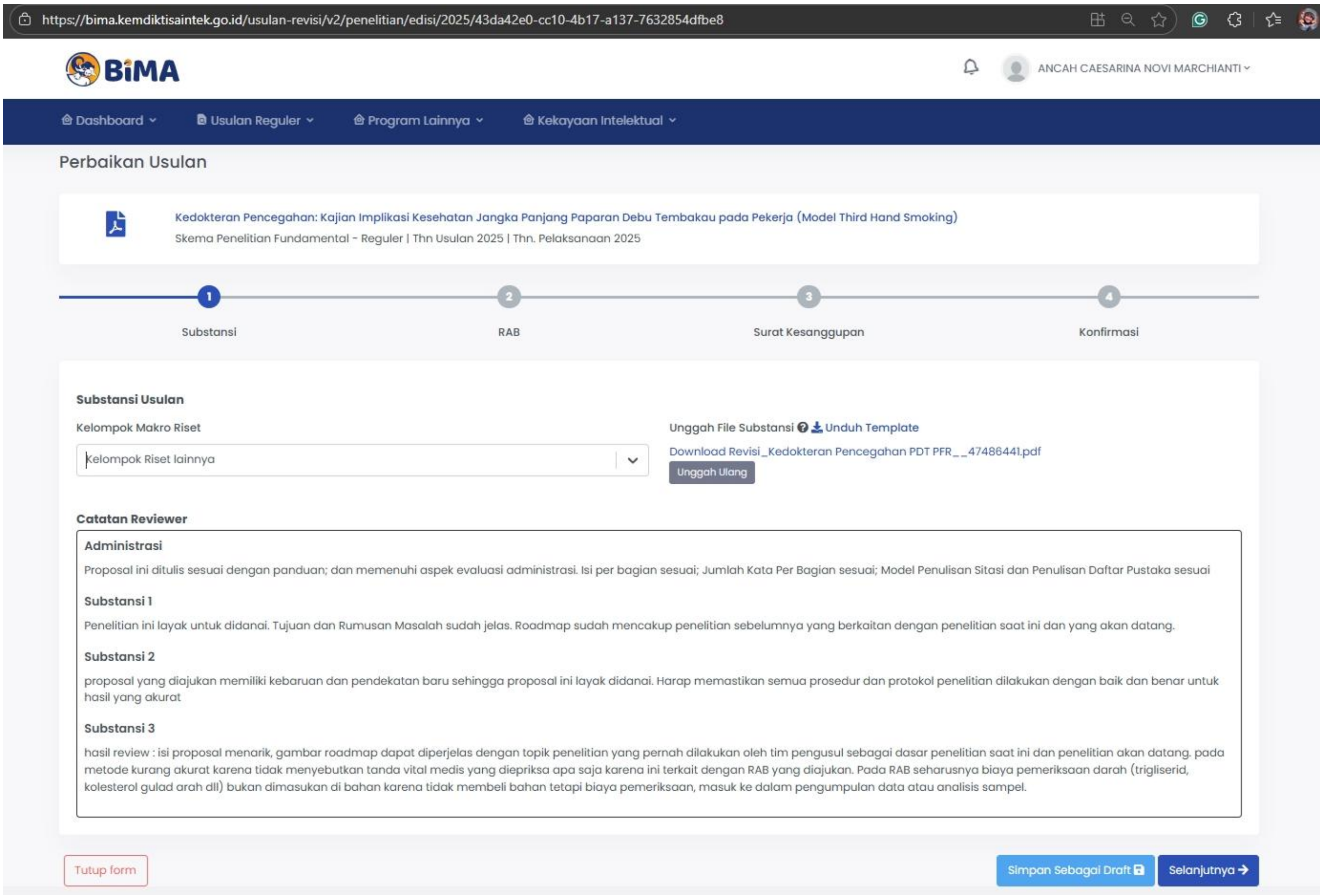

Reviewer	Revision points	Respond
Reviewer 1	Penelitian ini layak untuk didanai. Tujuan dan Rumusan Masalah sudah jelas. Roadmap sudah mencakup penelitian sebelumnya yang berkaitan dengan penelitian saat ini dan yang akan datang.<br><br><u>Translate</u><br>This research is eligible for funding. Objectives and Problem Formulation are clear. Roadmap included previous research related to current and future research.	-
Reviewer 2	proposal yang diajukan memiliki kebaruan dan pendekatan baru sehingga proposal ini layak didanai. Harap memastikan semua prosedur dan protokol penelitian dilakukan dengan baik dan benar untuk hasil yang akurat.<br><br><u>Translate</u>	All the procedures and research protocols will be carefully done and followed the board ethical committee recommendation.

	The proposal has novelty and a new approach so that this proposal eligible to be funded. Please ensure all procedures and research protocols are carried out properly and correctly for accurate results.	
Reviewer 3	hasil review : isi proposal menarik, gambar roadmap dapat diperjelas dengan topik penelitian yang pernah dilakukan oleh tim pengusul sebagai dasar penelitian saat ini dan penelitian akan datang. pada metode kurang akurat karena tidak menyebutkan tanda vital medis yang diepriksa apa saja karena ini terkait dengan RAB yang diajukan. Pada RAB seharusnya biaya pemeriksaan darah (trigliserid, kolesterol gulad arah dll) bukan dimasukan di bahan karena tidak membeli bahan tetapi biaya pemeriksaan, masuk ke dalam pengumpulan data atau analisis sampel.<br><br>Translate<br>Review results: the content of the proposal is interesting, the roadmap picture can be clarified with the research topics that have been carried out by the proposing team as the basis for current and future research. the method is less accurate because it does not mention what medical vital signs are examined because this is related to the proposed RAB (budget plan). In the RAB, the cost of blood tests (triglycerides, cholesterol, blood glucose, etc.) should not be included in the materials because they do not buy materials but the cost of the examination is included in data collection or sample analysis.	The method revised with a more complete explanation of each procedures/protocols. The RAB (budget plan) also revised according reviewer suggestions.

KEMENTERIAN PENDIDIKAN TINGGI, SAINS, DAN TEKNOLOGI
UNIVERSITAS JEMBER
FAKULTAS KEDOKTERAN
KOMITE ETIK PENELITIAN KESEHATAN (KEPK)
Jalan Kalimantan 37, KampusTegalBoto, Jember 68121
Telepon: (0331) 324446,337877, Faksimile: (0331) 324446
Laman: fk.unej.ac.id, Email: fk@unej.ac.id, Email: kepk.fk@unej.ac.id

KETERANGAN PERSETUJUAN ETIK
E T H I C A L A P P R O V A L

Nomor: 1578/UN25.1.10.2/KE/2025

Komisi Etik Fakultas Kedokteran Universitas Jember dalam upaya melindungi hak asasi dan kesejahteraan subyek penelitian kedokteran, telah mengkaji dengan teliti protokol berjudul :

The Ethics Committee of the Faculty of Medicine, Jember University, With regards of the protection of human rights and welfare in medical research, has carefully reviewed the proposal entitled :

Kedokteran Pencegahan : Kajian Implikasi Kesehatan Jangka Panjang Paparan Debu Tembakau pada Pekerja (Model Third Hand Smoking)

Baseline of Cohort Study: The Impact of Exposure to Industrial Tobacco Dust on Metabolic Parameter

Peneliti Utama<br>*Name of the principal investigator*	:	dr. Ancah Caesarina Novi Marchianti, Ph. D, FISPH, FISCM
NIM/NIP	:	198203092008122002
Nama Institusi<br>*Name of institution*		- Fakultas Kedokteran Universitas Jember<br>- Kagoshima University, Japan<br>- University of Occupational and Environmental Health, Japan<br>- National Institute for Minamata Disease, Japan<br>- Badan Riset dan Inovasi Nasional (BRIN)

Dan telah menyetujui protokol tersebut diatas.
And approved the above mentioned proposal.

Masa berlaku persetujuan etik ini 1 tahun
The validity period of this ethical approval is 1 year

Jember, 11 JUN 2025
Ketua Komisi Etik Penelitian Kesehatan

dr. Ayu Munawaroh Aziz, M.Biomed
NIP. 198903132014042002

Tanggapan Anggota Komisi Etik

Peneliti	:	dr. Ancah Caesarina Novi Marchianti, Ph. D, FISPH, FISCM
NIM/NIP	:	198203092008122002
Judul Penelitian	:	*Baseline of Cohort Study: The Impact of Exposure to Industrial Tobacco Dust on Metabolic Parameter*

***Review protokol etik* :**

Berdasarkan pertimbangan 3 prinsip etika, 7 standar, dan 25 butir pedoman etik penelitian pada manusia oleh CIOMS-WHO. Serta berdasarkan pedoman Guide For The Care And Use Of Laboratory Animal (1996). Maka pertimbangan etik untuk penelitian dengan judul tersebut diatas adalah:

1. Peneliti harus memastikan keamanan lingkungan ketika menguji sampel penelitian di lingkungan yang berhubungan langsung dengan masyarakat.
2. Penjelasan informed consent kepada subjek/responden harus jelas, menggunakan bahasa yang mudah dimengerti oleh subjek/responden.
3. Peneliti harus memberikan tali asih sebagai bentuk penghargaan kepada subjek penelitian
4. Peneliti harus menjaga rahasia subjek penelitian dan menyesuaikan dengan situasi di tempat pelayanan saat mengambil data penelitian.
5. Peneliti harus memberikan bantuan pengobatan kepada subyek penelitian apabila ditemukan penyakit akibat paparan tembakau.

Kesimpulan: Penelitian dapat dilanjutkan dengan syarat mematuhi pertimbangan etik tersebut diatas.

Jember, ...
Ketua Komisi Etik Penelitian Kesehatan

KOMISI ETIK PENELITIAN KESEHATAN FAKULTAS KEDOKTERAN UNIVERSITAS JEMBER

dr. Ayu Munawaroh Aziz, M.Biomed
NIP. 198903132014042002
